# Injectable hyaluronic-acid-doxycycline hydrogel therapy in experimental rabbit osteoarthritis

**DOI:** 10.1186/1746-6148-9-68

**Published:** 2013-04-10

**Authors:** Hsien-Tsung Lu, Ming-Thau Sheu, Yung-Feng Lin, Jai Lan, Yi-Ping Chin, Ming-Shium Hsieh, Chao-Wen Cheng, Chien-Ho Chen

**Affiliations:** 1 Graduate Institute of Clinical Medicine, College of Medicine, Taipei Medical University, No. 250 Wu-Hsing Street, , Taipei City, Xinyi District, 110, Taiwan; 2 School of Pharmacy, College of Pharmacy, Taipei Medical University, No. 250 Wu-Hsing Street, , Taipei City, Xinyi District, 110, Taiwan; 3 Department of Medical Technology, School of Medical Laboratory Science & Biotechnology, Taipei Medical University, No. 250 Wu-Hsing Street, , Taipei City, Xinyi District, 110, Taiwan; 4 Department of Microbiology and Immunology, School of Medicine, Taipei Medical University, No. 250 Wu-Hsing Street, , Taipei City, Xinyi District, 110, Taiwan; 5 Department of Orthopedics and Traumatology, Taipei Medical University Hospital, No. 250 Wu-Hsing Street, , Taipei City, Xinyi District, 110, Taiwan

**Keywords:** Osteoarthritis, Disease-modifying osteoarthritis drugs, Connective tissue structure-modifying agents, Hyaluronic acid, Doxycycline

## Abstract

**Background:**

Osteoarthritis (OA) is a common joint disease that causes disabilities in elderly adults. However, few long-lasting pharmacotherapeutic agents with low side effects have been developed to treat OA. We evaluated the therapeutic effects of intra-articular injections of hydrogels containing hyaluronic acid (HA) and doxycycline (DOX) in a rabbit OA model.

**Results:**

Thirteen week old New Zealand White rabbits undergone a partial meniscectomy and unilateral fibular ligament transection were administered with either normal saline (NT), HA, DOX or HA-DOX hydrogels on day 0, 3, 6, 9 and 12; animals were also examined the pain assessment in every three days. The joint samples were taken at day 14 post-surgery for further histopathological evaluation. The degree of pain was significantly attenuated after day 7 post-treatment with both HA and HA-DOX hydrogels. In macroscopic appearance, HA-DOX hydrogel group showed a smoother cartilage surface, no or minimal signs of ulceration, smaller osteophytes, and less fissure formation in compare to HA or DOX treatment alone. In the areas with slight OA changes, HA-DOX hydrogel group exhibited normal distribution of chondrocytes, indicating the existence of cartilage regeneration. In addition, HA-DOX hydrogels also ameliorated the progression of OA by protecting the injury of articular cartilage layer and restoring the elastoviscosity.

**Conclusion:**

Overall, from both macroscopic and microscopic data of this study indicate the injectable HA-DOX hydrogels presented as a long-lasting pharmacotherapeutic agent to apply for OA therapy.

## Background

Osteoarthritis (OA) is a chronic and multifaceted degenerative joint disease in which the articular cartilage and the surrounding extracellular matrix (ECM) are destroyed [1,2]. It is associated with increasing age because the articular cartilage of the joints may degrade with continual wear. An imbalance between the repair and degradation of the cartilage may disrupt the collagen matrix, resulting in OA. Non-steroidal anti-inflammatory drugs, calcitonin, and glucosamine have been used to treat OA [3]. However, these agents either have serious side effects or may not be ideal for long-term therapy. Recent studies of OA therapeutics have focused primarily on the development of disease-modifying osteoarthritis drugs (DMOADs) and connective tissue structure-modifying agents (CTSMAs) [4-7].

Doxycycline (DOX) has been used to treat the symptoms of OA [8-11]. The synthesis of inducible nitric oxide synthase is inhibited by DOX, which suppresses the secretion of matrix metalloproteinases (MMPs) by chondrocytes, thus relieving the degradation of type II collagen and aggrecan. In addition, DOX significantly suppresses the production of inflammatory cytokines, such as interleukin-1 (α/β) and interleukin-6, which inhibits inflammation in OA synovial cells and chondrocytes [12-15]. In an anterior-cruciate-ligament rupture-induced spontaneous OA model, DOX significantly improved the structure of the subchondral bone [16]. Clinical research has also shown that DOX can slow the rate of joint-space narrowing in the knees with established OA [14]. Thus, the chondroprotective effects of the suppression of catabolic cytokine cascades by DOX treatment may represent the ideal properties of both DMOAD- and CTSMA-based therapies for OA.

Hyaluronic acid (HA) occurs naturally in the ECM and synovial fluid. Imbalances in HA stability can result in the development of OA, and the joints of OA patients have been shown to contain shorter HA fragments than those found in normal joints [17,18]. The clinical outcomes of intra-articular HA and derived products had been critically reviewed, and proven to be an effective, safe, and tolerable treatment for knee OA [19]. Intra-articular injections of HA and other lubricating substances have been shown to relieve OA symptoms by alleviating pain and irritation [20,21]. Inflammation reduces the viscoelasticity of synovial fluid, and intra-articular injections of HA can compensate for the loss of joint lubrication by adhering to the cartilage surfaces and protecting them from damage [22-24]. It has been reported that nonmodified HA only with a half-life of 10–13 h, while chemically modified HA product, such as hylan G-F 20, was lasting to 8.8 ± 0.9 days [25,26]. The rapid clearance and elimination of HA intra-articular injection may limit its clinical usage; therefore, the improvements of longer residence time may extend the therapeutic benefit and clinical applications.

We hypothesized that the combination of HA and DOX in a hydrogel [27] might produce additive effects in OA therapy through the anti-inflammatory and analgesic effects of DOX and HA, respectively, and the increased viscoelasticity of the synovial fluid resulting from the high-molecular-weight hydrogel polymer. In addition, the slow release of DOX from the hydrogel polymer may sustain its therapeutic effects. We produced an injectable HA-DOX hydrogel, and evaluated its efficacy as an intra-articular treatment in a rabbit model of OA.

## Results

### Rheological characteristics of the HA-DOX hydrogels

The mixture of HA, DOX, and ZnCl in an aqueous solution produced a thermo-reversible, water-soluble hydrogel. Because it had been previously reported that the higher-molecular-weight, more viscoelastic hylan G-F 20 has significantly greater pain-relieving properties than does the lower-molecular-weight, less-viscoelastic HA [24], the rheological characteristics (G’, dynamic elastic modulus and G”, dynamic viscous modulus) of the HA-DOX hydrogel and an HA-DOX mixture without zinc cations were assessed over a range of DOX concentrations (Table 1).

**Table 1:** **Rheological characteristics of hydrogels containing hyaluronic acid (HA) and various concentrations of doxycycline (DOX) with Zn**^**2+ **^**and HA-DOX solutions without Zn**^**2+**^

	**G’ (Pa)**				**G” (Pa)**			
	**0.01 Hz**	**0.1 Hz**	**0.5 Hz**	**2.5 Hz**	**0.01 Hz**	**0.1 Hz**	**0.5 Hz**	**2.5 Hz**
HA^#^	0.020	0.056	0.435	7.056	0.071	0.763	4.160	13.170
Hydrogel 25^*^	0.055	0.238	1.761	8.473	0.093	0.801	4.749	13.716
Hydrogel 50^*^	0.052	0.347	2.302	8.574	0.261	0.839	4.910	13.970
Hydrogel 100^*^	0.338	1.166	2.796	10.363	1.087	2.055	6.492	16.895
Solution 25^*^	0.001	0.053	1.941	3.721	0.045	0.602	3.846	9.636
Solution 50^*^	0.004	0.187	1.263	3.969	0.049	0.489	3.160	10.004
Solution 100^*^	0.003	0.012	1.202	2.750	08.041	0.487	3.691	10.474

Note: 0.5 Hz is the approximate frequency corresponding to the movement of the knee joint in walking; G’, dynamic elastic modulus; G”, dynamic viscous modulus.

Hydrogel, containing 10 mg/mL HA and indicated concentrations of DOX with Zn^2+^; Solution, containing 10 mg/mL HA and indicated concentrations of DOX without Zn^2+^.

^#^Concentration of HA = 10 mg/mL.

^*^Different concentration of DOX (i.e. 25, 50,100) in μg/mL.

2.5 Hz is the approximate frequency corresponding to the movement of the knee joint in running.

In the absence of zinc cations, increasing concentrations of DOX caused progressive decreases in both the G’ and the G” as the HA and DOX precipitated because of charge interactions. Compared with the HA, both the G’ and G” were higher for the HA-DOX hydrogel, with the G’ and the G” increasing with DOX concentrations in the presence of zinc cations, indicating that ionic bonding between the HA and the DOX molecules had occurred within the high-MW polymer. Moreover, as the oscillating frequency decreased, both the G’ and the G” decreased more slowly as the DOX concentration was increased, which further indicated that a high degree of DOX cross-linking had occurred. We concluded that the viscoelasticity of the HA-DOX hydrogel was comparable to or greater than that of the HA.

### HA-DOX hydrogel exhibited low cytotoxicity *in vitro*

**Figure 1:**
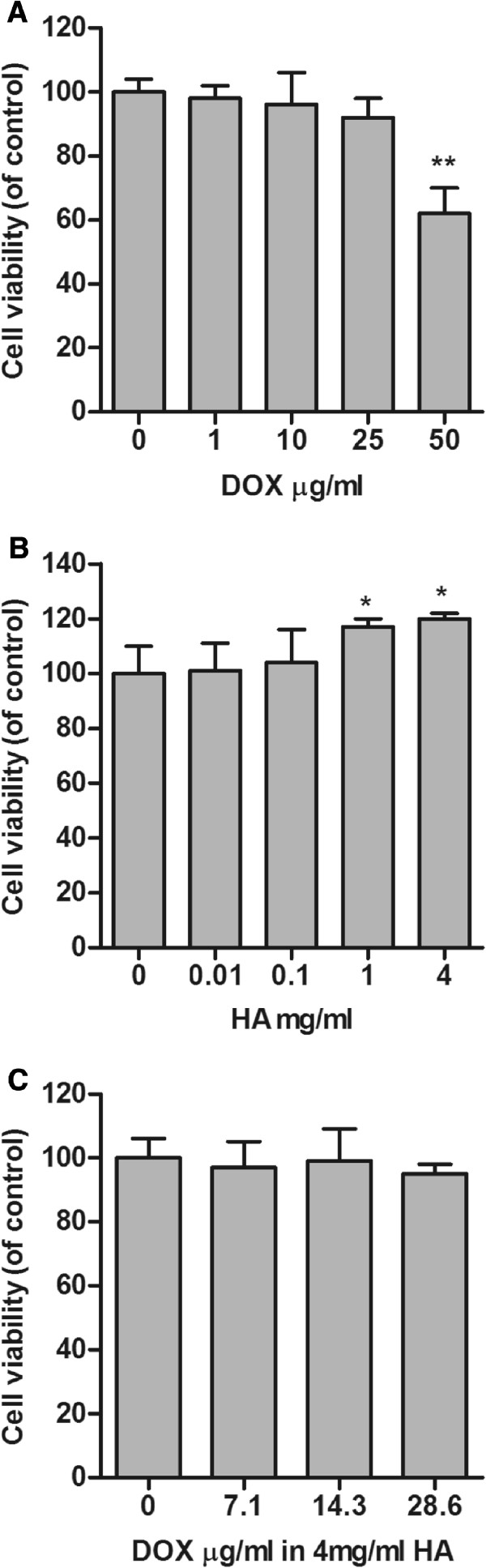
**HA-DOX hydrogel showed low cytotoxicity.** The influence of DOX (**A**), HA (**B**), and the HA-DOX hydrogel (**C**) on the survival of human chondrosarcoma cells SW1353 was assessed in 3 independent experiments using a tetrazolium-based cell viability assay. The relative number of surviving cells were quantified and normalized to that of the NT group, which represented 100% survival (*p < 0.05, **p < 0.01).

As shown in Figure 1A, SW1353 cell viability was not noticeably influenced by 0.01 to 25 μg/mL DOX, whereas 50 μg/mL DOX significantly inhibited the survival of SW1353 cells. Treatments of SW1353 cells with 0.01, 0.1, 1.0, and 4.0 mg/mL HA for 24 h showed no cytotoxicity (Figure 1B). Treatments with HA-DOX hydrogels containing 4 mg/mL HA and 0.0, 7.15, 14.3, or 28.6 μg/mL DOX for 24 h did not reduce SW1353 cell viability or alter cell morphology (Figure 1C).

### Intra-articular HA-DOX hydrogel injections reduced pain

**Figure 2:**
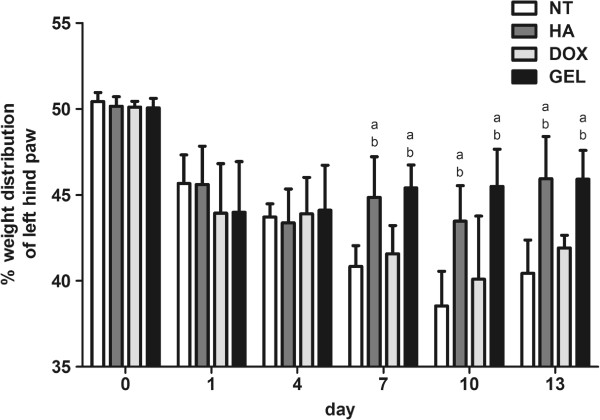
**Intra-articular HA-DOX hydrogel presented relieve the pain.** Influence of intra-articular injection of NT, HA, DOX and HA-DOX hydrogel groups on the left-hind paw percent-weight distribution. The results were compared to evaluate differences between the NT group (p < 0.05, a) and the DOX group (p < 0.05, b).

Following the surgical induction of OA, the percentage weight distribution of the left-hind paw decreased significantly in each group (Figure 2). The percentage weight distribution values for both the NT and the DOX groups decreased over the course of the experiment. In the HA and HA-DOX hydrogel groups, the percentage weight distributions showed gradual and significant increases on 7, 10, and 13 d after surgery, compared with the NT and DOX groups (Figure 2), indicating that the intra-articular injection of the HA and the HA-DOX hydrogel treatments exhibited analgesic effects.

### Intra-articular HA-DOX hydrogel injections reduced OA pathology

**Figure 3:**
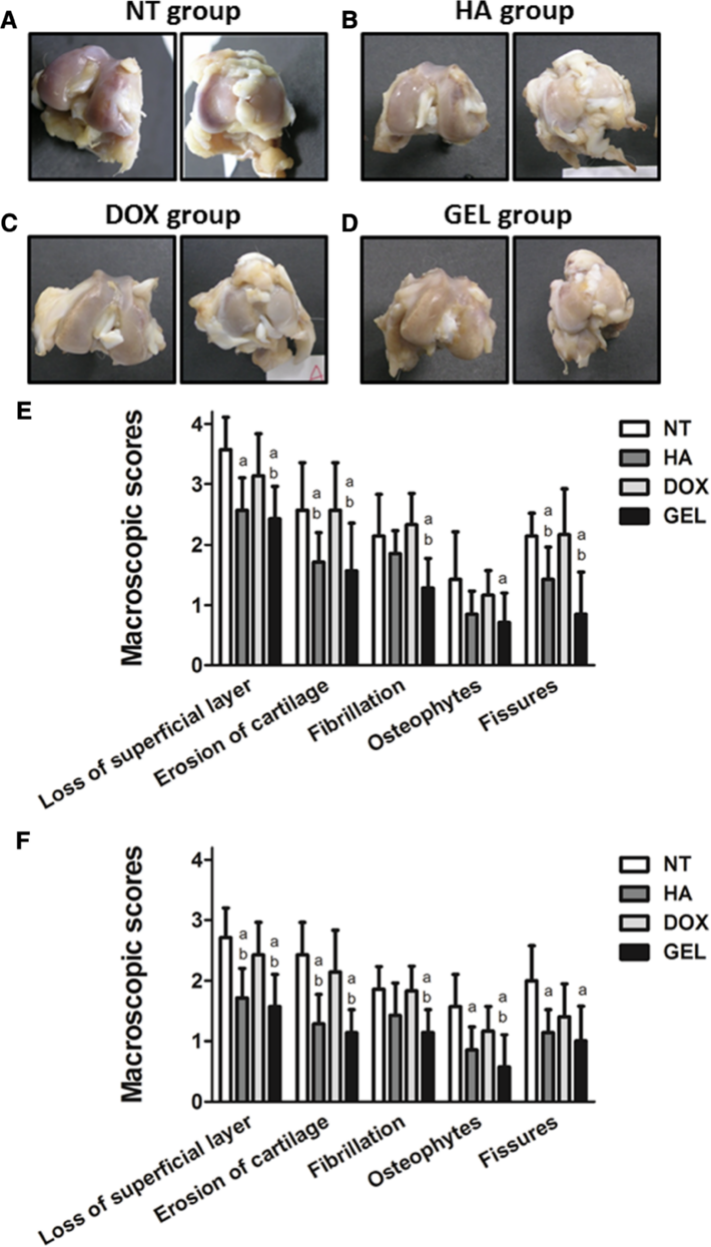
**Intra-articular HA-DOX hydrogel injections reduced the macroscopic appearance of OA pathology.** Macroscopic appearance of the articular surfaces of the femoral condyles (left panels) and the tibia plateau (right panels) of the NT (**A**), HA (**B**), DOX (**C**), and HA-DOX hydrogel groups (**D**). In addition, the femur (**E**) and the tibia (**F**) samples were examined using surgical magnifying glasses to evaluate the damage to the articular cartilage surfaces and the various parameters were scored as described in the Materials and methods. The results were compared to evaluate differences between the NT group (p < 0.05, a) and the DOX group (p < 0.05, b).

The macroscopic examination of the specimens showed that the articular cartilage was rough and dull on both the femoral and tibial surfaces. The most remarkable damage in each group occurred at the femoral condyle, the femoral trochlea, and the tibial plateau. The NT group had the highest lesion scores for all parameters among the various treatment groups, and the HA-DOX hydrogel group had significantly lower scores for all parameters (Figure 3). Although the differences between the HA and the HA-DOX hydrogel groups were not statistically significant for all OA features examined, lower lesion scores for the loss of the superficial layer of the femur, the presence of fibrillation, and the presence of osteophytes of the femur and the tibia were recorded for the HA-DOX hydrogel group. Overall, the intra-articular injection of the HA-DOX hydrogel significantly inhibited the progression of OA in the rabbit model.

**Figure 4:**
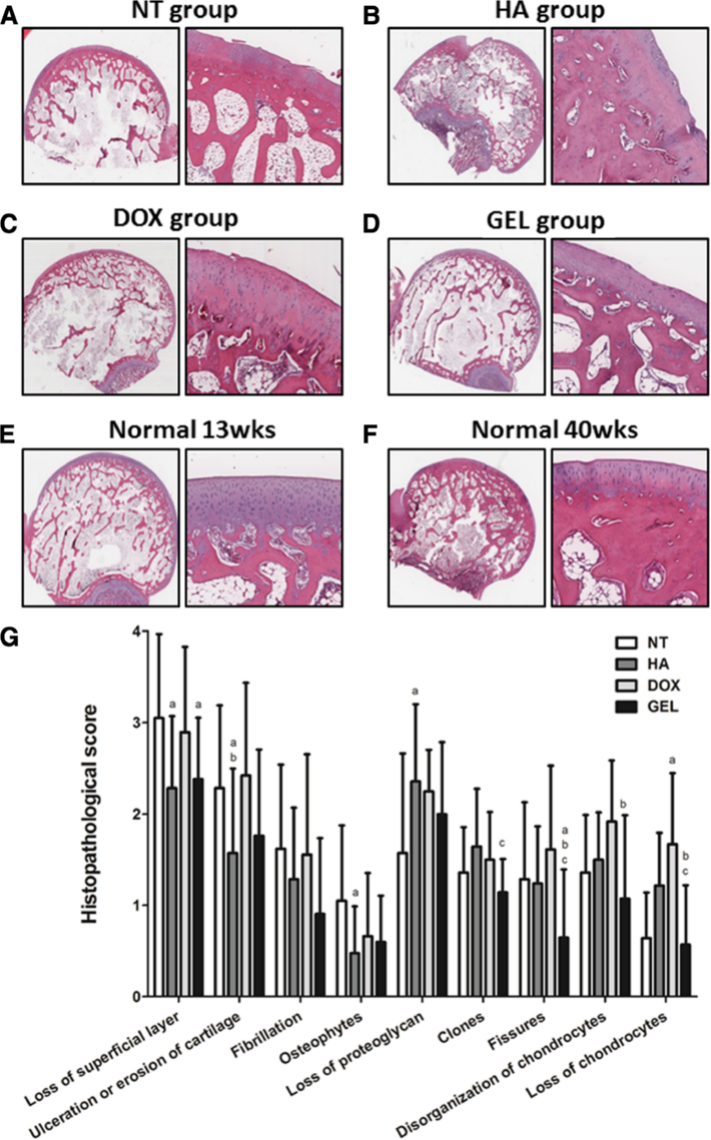
**Intra-articular HA-DOX hydrogel injections reduced the attenuated microscopic appearance of femoral condyles in OA.** Histological examinations of hematoxylin and eosin stained sections of the cartilage in the femoral condyles at 150X (left panels) and 300X (right panels) magnifications. The samples from the groups were treated with NT (**A**), HA (**B**), DOX (**C**), and HA-DOX hydrogel groups (**D**) separately. The normal-young group (13-week-old rabbits) (**E**) and the normal-old group (40-week-old rabbits) (**F**) did not receive intra-articular treatments. The various features of OA pathology were scored on a scale of 0 to 4, with 4 representing OA lesions of the worst possible severity (**G**), as described in the Materials and methods. The results were compared to evaluate differences between the NT group (p < 0.05, a); the DOX group (p < 0.05, b); and the HA group (p < 0.05, c).

**Figure 5:**
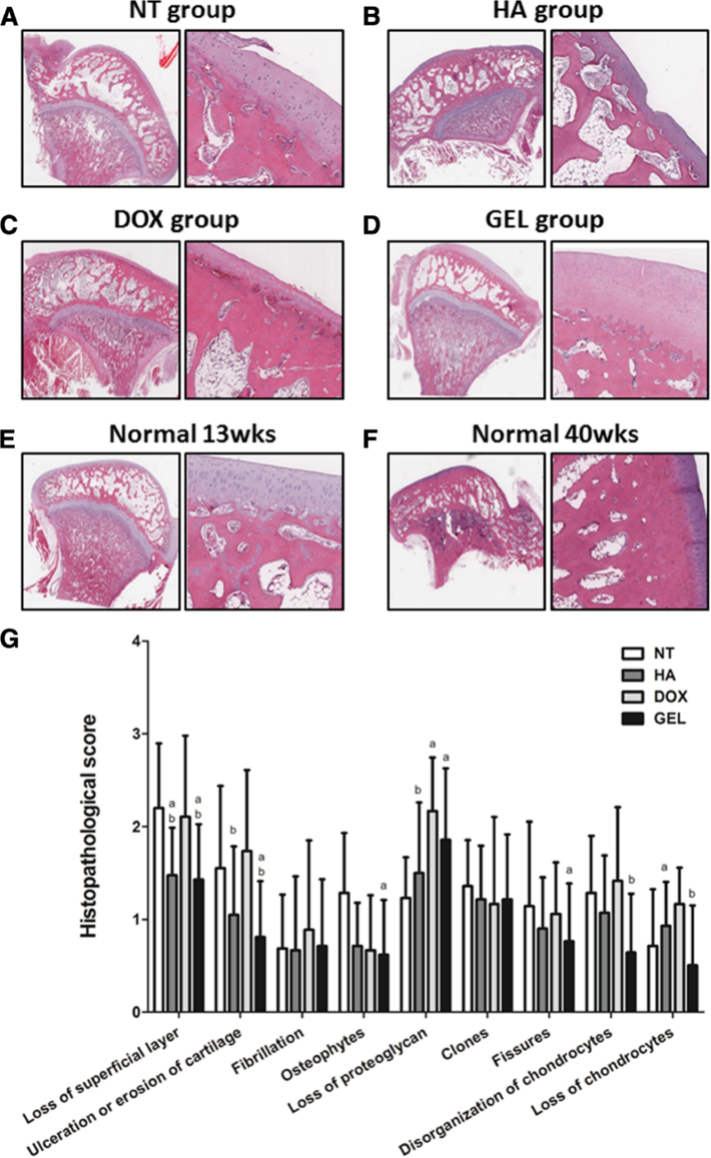
**Intra-articular HA-DOX hydrogel injections reduced the attenuated microscopic appearance of tibial plateau in OA.** Histological examinations of hematoxylin and eosin stained sections of the cartilage in the tibial plateau at 150X (left panels) and 300X (right panels) magnifications. The samples from the NT (**A**), HA (**B**), DOX (**C**), and HA-DOX hydrogel groups (**D**) were processed separately. The normal-young group (13-week-old rabbits) (**E**) and the normal-old group (40-week-old rabbits) (**F**) did not receive intra-articular treatments. The various features of OA pathology were scored on a scale of 0 to 4, with 4 representing OA lesions of the worst possible severity (**G**), as described in the Materials and methods. The results were compared to evaluate differences between the NT group (p < 0.05, a); the DOX group (p < 0.05, b); and the HA group (p < 0.05, c).

**Figure 6:**
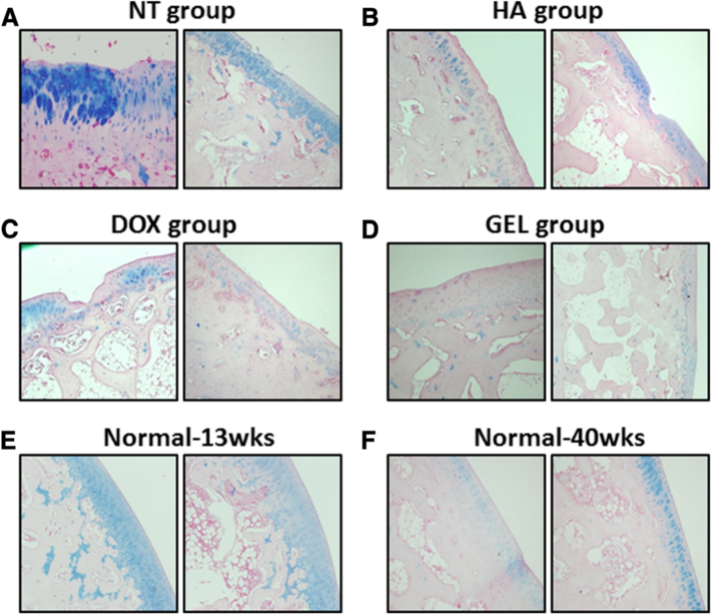
**The expression pattern of proteoglycan in the microscopic appearance of OA pathology.** Histological examination of Alcian-blue stained sections of cartilage from the femoral condyles (left panels) and the tibial plateau (right panels) at 300 X magnifications. The samples from the NT (**A**), HA (**B**), DOX (**C**), and HA-DOX hydrogel groups (**D**) were processed separately. The normal-young group (13-week-old rabbits) (**E**) and the normal-old group (40-week-old rabbits) (**F**) did not receive intra-articular treatments.

The microscopic histological examination showed that the intra-articular injection of the HA-DOX hydrogel reduced the loss of chondrocytes at the femoral condyles (Figure 4) and at the tibial plateau (Figure 5). Treatment with either the HA-DOX hydrogel or the HA attenuated lesion formation. The NT group had many chondrocytes, whereas the DOX group had few. The clone parameter (cluster formation) revealed a typical feedback situation. The HA-DOX hydrogel group had the fewest chondrocyte clones, whereas the DOX, HA, and NT groups showed significant cloning of chondrocytes. The NT group showed the lowest loss of proteoglycan. Both the HA and the HA-DOX hydrogel groups showed reduced losses of the superficial layer, compared with the DOX and NT groups (Figure 6). Similar results were observed for ulceration, fibrillation, osteophytes, fissures, and disorganization of chondrocytes, with the HA and the HA-DOX hydrogel groups displaying significantly lower scores for most of the parameters. Overall, the HA-DOX hydrogel treatment resulted in the greatest reductions in OA pathology.

## Discussion

In our study, the HA-DOX hydrogel was formed by polymerizing electrostatic interactions between HA and DOX molecules, followed by cross-linking through the phenolic moieties of the immobilized DOX by zinc-mediated chelation. Increasing DOX concentrations in the hydrogel exhibited increased G’ and G” values for the polymer, with the higher DOX concentrations resulting in a more highly cross-linked structure that displayed greater viscosity and viscoelasticity. Our results show that the enhanced rheological properties of the HA-DOX hydrogel were beneficial for intra-articular applications in OA therapy.

When applied intra-articularly, HA covers the surface of the cartilage, acting as a cushion that absorbs pressure and vibration and prevents further erosion. The highly viscous HA has been used in intra-articular injections to reverse the loss of viscosity and viscoelasticity of the synovial fluid resulting from inflammation and physical wear [21]. Our data show that the HA-DOX hydrogel possesses higher viscosity and viscoelasticity than those of HA alone, suggesting that it may provide better chondroprotective effects (Table 1). The cytotoxicity evaluation showed that treatment with high concentrations of DOX alone (50 μg/mL) was toxic to the SW1353 chondrosarcoma cells. However, the HA-DOX hydrogel displayed significantly lower cytotoxicity, compared with similar concentrations of DOX (Figure 1). These results suggest that the HA-DOX hydrogel may maintain protective effects for longer periods than those of either HA or DOX used alone.

Intra-articular application of HA promotes the nutrient transport and waste excretion functions of synovial fluid, and covers the articular surfaces to protect the cartilage. The adherence of HA to articular surfaces has been reported to protect nerve endings that may be exposed by thinner, degraded cartilage [28,29]. The pain-relieving property of HA is an important disease-modifying quality for clinical purposes. Thus, the increase in viscosity and viscoelasticity of the HA-DOX hydrogel indicates that it should produce similar effects. In a prospective cohort study to evaluate pain and functional outcomes from OA patients with intra-articular HA injection over 6 months, HA was associated with lower functional pain severity, which represented an increase in the quality of the movement and functional activity [30]. Another study had also reported the mid-term efficacy (2-year) of intra-articular HA injection improve the joint structure and relieve pain in patients with knee OA [31]. In our *in vivo* experiments, the percentage weight distributions of the injured hind paw were used as an indicator of the analgesic effects of the treatments (Figure 2). The results showed a significant analgesic effect following intra-articular injection of the HA and the HA-DOX hydrogel, compared with the DOX and NT treatments, on Days 7, 10, and 13 following the induction of OA and the initiation of treatment. This indicates the HA-DOX hydrogel can still retain the pain-relieving property of HA; however, its long-term effect of pain-reliving need be further studied.

The macroscopic examination revealed that both the HA and the HA-DOX hydrogel treatments diminished OA features, compared with the NT and DOX treatments. Moreover, the HA-DOX hydrogel group exhibited greater therapeutic effects than those observed in the HA group. However, intra-articular injection of DOX alone did not abrogate the OA pathology, compared with the results observed in the NT group (Figures 3, 4, and 5). Histopathological findings (Figures 4 and 5) mirrored the results of the macroscopic evaluation and pain assessment. Both the HA and the HA-DOX hydrogel treatments reduced cartilage degradation at the femoral condyles and the tibial plateau, and effectively reduced the loss of the superficial layer, ulceration, the production of osteophytes, the creation of fissures, and the disorganization of cartilage, compared with the NT and DOX treatments (Figures 4 and 5). As the results of macroscopic examination, the chondroprotective effects of HA-DOX hydrogel treatment were superior to those of the HA treatment. The therapeutic treatments had started immediately following surgery in this study that may limit the therapeutic outcomes interpreting. Intra-articular hylan G-F 20 injection in 4 weeks post-operatively to the same model had also been reported the protective effects in maintains cartilage integrity and decreases osteophyte formation [32]. This may imply the potential of the HA-DOX hydrogel in application to OA models with the onset of symptoms. Future studies with different therapeutic regimens of HA-DOX hydrogel could provide additional support to our current findings.

The loss of chondrocytes and of proteoglycan scores for the NT group was lower than that of the HA group and that of the HA and HA-DOX hydrogel groups, respectively. This may result in a feedback phenomenon, in which a greater amount of chondrocytes produced comparatively greater amounts of proteoglycan. However, since the NT group presented similar proteoglycan level to normal groups, it still cannot rule out the possibility of the adversary effects of proteoglycan depletion following the treatments. As erosion and loss of cartilage occurred, the remaining chondrocytes secreted more glycoprotein for bone regeneration, resulting in the production of osteophytes. The NT group had the highest score of osteophytes; this may further support the involvement of such feedback. The clone parameter assessed the aggregation of chondrocytes, which may also result from the feedback phenomenon. Aggregation results from abnormal cell proliferation following cartilage destruction or chondrocyte perturbations. Thus, clones can serve as signs of altered cartilage. However, the reliability of this inference has not been thoroughly established.

In addition to HA-DOX hydrogel, several different HA-derived combination compounds were recently reported the therapeutic effects by intra-articular injection in rabbit OA model. Celecoxib-loaded liposomes embedded in HA gel combination was more effective than a single drug in pain control and cartilage protection [33]. Intra-articular injection of collagen tripeptide, and collagen tripeptide and HA mixtures seemed to be effective for the initiation period of cartilage degeneration partly by promotion of type II collagen synthesis and prevention of proteoglycan loss [34]. These findings suggest the developing potential of HA-derived combination compounds in the OA therapy.

It is unlikely that the beneficial effects of the HA-DOX hydrogel treatment can be attributed to a temporary enhancement of the viscoelasticity and viscosity of the synovial fluid. Based on the demonstrated anti-inflammatory effects of DOX in clinical applications, the HA-DOX hydrogel therapy possibly produced disease-modifying biological actions that influenced the progression of OA in the rabbit model. Thus, the HA-DOX hydrogel represents an ideal DMOAD and CTSMA. The long-term therapeutic effects of intra-articular HA-DOX hydrogel treatment in chronic OA warrants further investigation.

## Conclusions

The HA-DOX hydrogel, composed of a polymer of HA and DOX with zinc cations, possessed higher viscosity and viscoelasticity, and provided better chondroprotective effects than those of an HA alone. Therefore, the injectable HA-DOX hydrogel may represent a desirable DMOAD for OA therapy.

## Methods

### Cells and reagents

The human chondrosarcoma cell line, SW1353, was obtained from American Type Culture Collection (Manassas, VA, USA). We used 10 mg/mL ARTZ-Dispo HA (Seikagaku, Tokyo, Japan) in all of our experiments. The ARTZ-Dispo HA has a weight-average molecular weight (MW) of 60 to 120 kDa and a viscosity-average MW of 1650 kDa. DOX and zinc chloride (ZnCl2) were purchased from Sigma (St Louis, MO, USA), and the normal saline was provided by the Sin-Tong Company (Taoyuan, Taiwan).

### Hydrogel preparation and characterization

The HA-DOX hydrogels were prepared as previously described [27], except that we replaced the magnesium ions with zinc ions (2:1 DOX to Zn2 + ratio). The pH of the HA, ZnCl2, and DOX aqueous solutions were adjusted to 7.0 with a 50 mM phosphate buffer. The solutions were dispensed and cooled overnight in a refrigerator to facilitate polymerization. The resulting hydrogels were stored in a refrigerator and used within 1 d. The viscoelastic properties of the hydrogels were measured using a Haake RheoStress 1 rotation rheometer (Thermo Fisher, Waltham, MA, USA). The dynamic shear moduli, G’ (elasticity) and G” (viscosity), were determined using the oscillation mode with a frequency range of 0.01 to 2.5 Hz.

### Cell viability

Following the DOX, HA, and HA-DOX hydrogel treatments, the SW1353 cell viability was determined using a tetrazolium-based colorimetric (MTT) assay. Detergent was used to dissolve the formazan crystals in wells before directly measuring the absorbance at 570 nm with a spectrophotometer.

### Experimental OA model

All our animal protocols were approved by the Experimental Animal Review Committee of Taipei Medical University. Thirteen-week-old male New Zealand white rabbits were used for our experimental OA model according to a modified Colombo method and a modified score [35]. The *in vivo* animal experiments were performed using aseptic techniques. The rabbits were anesthetized using Zoletil (Zoletil-Virbac, Carros, France) and Rompun (Bayer, Leverkusen, Germany). The left knee joint was incised approximately 2 cm down the lateral aspect of the patella to expose and cut the lateral-collateral ligament (fibular ligament). The end of the popliteus tendon was incised to expose the lateral meniscus, and the lateral meniscus was resected in the midsection for 3.0 to 4.0 mm to detach approximately one-third of the meniscus. The subdermal muscular layer and skin were closed using knotted absorbable sutures and nylon sutures. A 0.22 mL injection of enrofloxacin (China Chemical and Pharmaceutical, Hsinchu, Taiwan) was administered subdermally near the thigh to avoid suppuration. A partial meniscectomy was performed immediately before the administration of the test drugs. The animals were housed individually in 350 × 527 × 350 mm (W × D × H) steel cages at 22 ± 3°C and 55% ± 20% humidity. Animals were fed RC4 pellet-type laboratory-animal food with no additional calcium supplement through a stainless steel pellet feeder, and tap water was provided continually.

### Experimental design

Following the partial meniscectomy of the left knee joint, the rabbits were divided into the NT (n = 8), HA (n = 8), DOX (n = 7), and HA-DOX GEL (n = 8) study groups, and received 0.2 mL intra-articular injections of normal saline, DOX (87.5 μg/mL), HA (10 mg/mL), or the HA-DOX hydrogel (10 mg/mL HA and 87.5 μg/mL DOX), respectively, to the meniscetomized left cavity of the knee joint using a 1 mL syringe with a 26G hypodermic needle immediately following surgery. The injections were repeated on 3, 6, 9, and 12 d following the meniscectomy.

### Pain assessment

Triplicate measurements of the hind-paw weight distributions were recorded using 2 scales that independently measured the weight borne by each hind paw. The mean percentage (%) weight distribution of the left-hind paw was calculated 1 d before surgery and on 1, 4, 7, and 13 d after surgery according to the following equation [36]:

$$\begin{array}{c} \%\;\mathrm{weight}\;\mathrm{distribution}\;\mathrm{of}\;\mathrm{left}\;\mathrm{hind}\;\mathrm{paw}\\ =\left(\frac{\mathrm{left}\;\mathrm{weight}}{\left(\mathrm{right}\;\mathrm{weight}\;+\;\mathrm{left}\;\mathrm{weight}\right)}\right)\times \;100 \end{array}$$

### Specimen collection

After the rabbits were euthanized, treated knee joint specimens were collected by osteotomy 3 cm above and below the knee joint. The specimens were fixed in 10% buffered formalin for 24 h. Fixed specimens were cleared of soft tissues and ligaments to allow the gross examination of the articular surfaces of the femoral condyles and the tibial plateau for the scoring of the features of OA pathology.

### Macroscopic and microscopic specimen examinations

The macroscopic examination of the specimens was performed using a surgical magnifying glass to evaluate the OA progression based on a modification of the parameters described by Colombo et al. (1983b). The loss of the superficial layer, the cartilage erosion, the loss of the cartilage luster, the presence of osteophytes, and the presence of fissures were evaluated based on the location, type, and size of the pathological feature. Digital photographs of the articular surfaces were recorded.

Histological sections were taken at both femoral condyles, the femoral trochlea, the internal tibial plateau, and the lateral tibial plateau. The specimens were separately stained with hematoxylin and eosin (H&E), periodic acid Schiff stain, and Alcian blue stain. In the microscopic examination, 9 histological parameters were scored based on a previously described grading system [37] as follows: loss of the superficial layer, the presence of ulceration or erosion (surface fragmentation), the presence of fibrillation, the presence of fissures (V-shaped clefts), the presence of osteophytes and/or chondrophytes, the loss of stainable proteoglycan, the disorganization of chondrocytes, the presence of clones, and the loss of chondrocytes.

Every histological parameter was scored on a scale of 0 to 4 according to the severity of the OA, by which cartilage with an appearance that was identical to healthy cartilage at the same age was graded as 0, and OA lesions of the worst possible severity were scored as 4. Cartilage thickness was microscopically measured at 10X magnification in increments of 0.01 mm of actual thickness, where 1 cm of the microscopic scale corresponded to 0.39 mm of actual thickness. The total score of all 5 parameters represented the severity of OA progression in each histological section. The sum of all scores from all histological sections of each animal represented the total score for that animal. The statistical analysis was based on the median and the mean of the sums of the total scores of all the animals in each treatment group.

### Statistical analysis

The data are expressed as the mean ± the standard deviation of the various measurements. The statistical significance of intergroup differences was analyzed using one-way analysis of variance (ANOVA) and the Duncan post-hoc test. Differences with a P value less than 0.05 were considered statistically significant.

## Competing interests

All authors declare that they have no competing interests.

## Authors’ contributions

HTL and designed the research and wrote the paper. MTS, YFL and JL performed the research and analyzed the data. MSH and YPC analyzed the data. CWC analyzed the data and wrote paper. CHC designed research and wrote paper. All authors read and approved the final manuscript.
